# Prediction of Chemical Respiratory Sensitizers Using GARD, a Novel *In Vitro* Assay Based on a Genomic Biomarker Signature

**DOI:** 10.1371/journal.pone.0118808

**Published:** 2015-03-11

**Authors:** Andy Forreryd, Henrik Johansson, Ann-Sofie Albrekt, Carl A. K. Borrebaeck, Malin Lindstedt

**Affiliations:** 1 Department of Immunotechnology, Lund University, Medicon Village, Lund, Sweden; 2 SenzaGen AB, Medicon Village, Lund, Sweden; National Institute of Health (NIH), UNITED STATES

## Abstract

**Background:**

Repeated exposure to certain low molecular weight (LMW) chemical compounds may result in development of allergic reactions in the skin or in the respiratory tract. In most cases, a certain LMW compound selectively sensitize the skin, giving rise to allergic contact dermatitis (ACD), or the respiratory tract, giving rise to occupational asthma (OA). To limit occurrence of allergic diseases, efforts are currently being made to develop predictive assays that accurately identify chemicals capable of inducing such reactions. However, while a few promising methods for prediction of skin sensitization have been described, to date no validated method, *in vitro* or *in vivo*, exists that is able to accurately classify chemicals as respiratory sensitizers.

**Results:**

Recently, we presented the *in vitro* based Genomic Allergen Rapid Detection (GARD) assay as a novel testing strategy for classification of skin sensitizing chemicals based on measurement of a genomic biomarker signature. We have expanded the applicability domain of the GARD assay to classify also respiratory sensitizers by identifying a separate biomarker signature containing 389 differentially regulated genes for respiratory sensitizers in comparison to non-respiratory sensitizers. By using an independent data set in combination with supervised machine learning, we validated the assay, showing that the identified genomic biomarker is able to accurately classify respiratory sensitizers.

**Conclusions:**

We have identified a genomic biomarker signature for classification of respiratory sensitizers. Combining this newly identified biomarker signature with our previously identified biomarker signature for classification of skin sensitizers, we have developed a novel *in vitro* testing strategy with a potent ability to predict both skin and respiratory sensitization in the same sample.

## Introduction

Respiratory sensitization is an allergic type I hypersensitivity reaction of the upper and lower respiratory tract caused by an immune response towards environmental proteins or certain low molecular weight (LMW) chemical compounds. Clinical symptoms of respiratory sensitization, including wheezing, bronchoconstriction and asthmatic attacks, develops in susceptible and previously sensitized individuals upon repeated exposure to the same compound [1]. Mechanistically, respiratory sensitization is initiated by the activation of CD4^+^ Th2 cells and mediated by the differentiation of B-lymphocytes through the increased production of allergen specific IgE antibodies [1–3].

While respiratory allergy is generally induced by protein allergens, LMW chemical compounds have primarily been associated with the induction of type IV hypersensitivity reactions involving CD8+ T cells and CD4+ Th1 cells and the onset of skin conditions such as Allergic Contact Dermatitis (ACD). However, certain classes of LMW chemical compounds, such as diisocyanates [4], acid anhydrides [5], platinium salts [6], reactive dyes [7], and chloramine T [8] may also sensitize the respiratory tract. Exposure to these LMW chemical compounds is generally limited to occupational settings, and repeated exposure during an extended period of time may eventually result in development of occupational asthma (OA) [3,9]. Although fewer chemicals are known to cause respiratory allergy (< 100 known substances) [10], compared to those causing contact dermatitis, health effects can still be disastrous. For example, acquired OA may result in chronic inflammation, airway hyperresponsiveness [11], extensive airway remodeling [12] and, thus, severely affecting the quality of life for affected individuals. The serious health effects associated with OA, as well as the introduction of new compounds into working environments (e.g cleaning agents and healthcare products [9,13–15]) highlights the need for accurate and reliable testing strategies for hazard classification of potential respiratory sensitizers. Proactive identification and characterization of these compounds thus remains an area of great importance.

A challenge in this respect, however, is that methods for risk assessment of chemicals inducing respiratory sensitization are greatly underdeveloped, with no validated assay to date [16]. Current approaches involve both *in-vivo* and *in-vitro* testing strategies. Until recently, the field has relied on animal based *in vivo* models. Among these animal based approaches, guinea pig testing [17], mouse IgE testing [18,19], rat Ig E testing [20,21] and mouse cytokine fingerprinting [22,23] have gained most attention. In addition, several murine models of chemically induced asthma, aiming at discriminating respiratory sensitizers from skin sensitizers, are described in the literature [24,25]. Although these approaches undoubtedly have contributed to the current understanding of the immunobiological mechanisms and cellular processes associated with development of respiratory sensitization, none of the methods have proven sufficiently reliable in order to be used as a routine assay for regulatory purposes. Additionally, there are considerable economical and ethical drawbacks associated with the use of animal based methods as screening tools.

Considerable efforts have therefore been made to develop cell-based *in-vitro* assays for sensitization of the respiratory tract, which correlates with the principle of the three Rs on reduction, refinement and replacement of animal experiments stated in Directive 201/63/EU[26]. Recent cell-based approaches have involved the use of single cell lines as models for different stages in the sensitization process, such as the dendritic cell line THP-1[27] and the epithelial cell lines BEAS-2B[28] and A549[29]. More advanced attempts to mimic the *in vivo* route of exposure to respiratory sensitizers have also been performed, using the commercially available MucilAir developed by Epithelix as a 3D cell model of human airway epithelium [30]. In addition, non-cell based *in silico* predictive models based on chemical reactivity is being explored within respiratory sensitization [31]. Despite ambitious attempts on the development of *in vitro* based methods for risk assessment of respiratory sensitization, no single method has yet proven reliable enough to be used for regulatory purposes [32,33].

In contrast to the lack of assays for respiratory sensitization, the literature describes several predictive models for identification of skin sensitizing chemicals (reviewed in [34]), with the animal based Local Lymph Node Assay (LLNA) [35] historically being the preferred method. Several *in vitro* models have also been described for the endpoint of skin sensitization, including the human Cell Line Activation Test (h-CLAT) [36,37], the Direct Peptide Activation Test (DRPA) [38] and the KeratinoSens test [39,40]. Recently, we also presented our in-house developed Genomic Allergen Rapid Detection (GARD) [41,42] *in vitro* assay as an accurate alternative to these methods. The GARD assay is based on measurements of transcriptional levels of a genomic biomarker signature (GARD Prediction Signature, or GPS) comprising 200 genes in the myeloid human cell line MUTZ-3 [43–45] using transcriptome-wide DNA microarray technology. The functionality of the GARD assay in terms of predictive performance was evaluated in a recent study using a cohort comprising 26 blinded compounds and 11 non-blinded compounds. The accuracy of the assay was estimated to 89% [46], which can be compared to 72% for the LLNA [47]. Skin sensitization models are interesting because it has been proposed that skin sensitization assays, such as LLNA, could be applied to classify also respiratory sensitizers [48,49]. The endpoint in the LLNA assay is the provoked proliferative response measured in the draining lymph node upon topical exposure of mice to a test chemical [50,51]. However, this proliferative response is induced by both respiratory sensitizers and skin sensitizers. Consequently, while the LLNA can be used for stratification of sensitizers from non-sensitizers, it is unable to accurately discriminate between skin sensitizers and respiratory sensitizers [1].

In this study, we present a human cell based testing strategy for assessment of respiratory sensitizers based on a genomic biomarker signature, as a novel *in-vitro* alternative to animal testing. We utilized the great versatility that comes with analyzing the complete transcriptome of cells and extended the concept of the GARD assay to include prediction of respiratory sensitizers by identification of a separate genomic biomarker signature, called the GARD Respiratory Prediction Signature (GRPS). We now present results that demonstrate that the GRPS can be used in order to classify respiratory sensitizers. The intended use of the identified biomarker signature will be in combination with GPS for classification of skin sensitizing chemicals. Thus, the GARD concept demonstrates a unique opportunity for a test platform that can simultaneously be used for risk assessment and hazard classification of both skin and respiratory sensitizing properties of unknown chemicals.

## Results

### Phenotypic analysis of unstimulated and chemically stimulated MUTZ-3 cells

**Fig 1:**
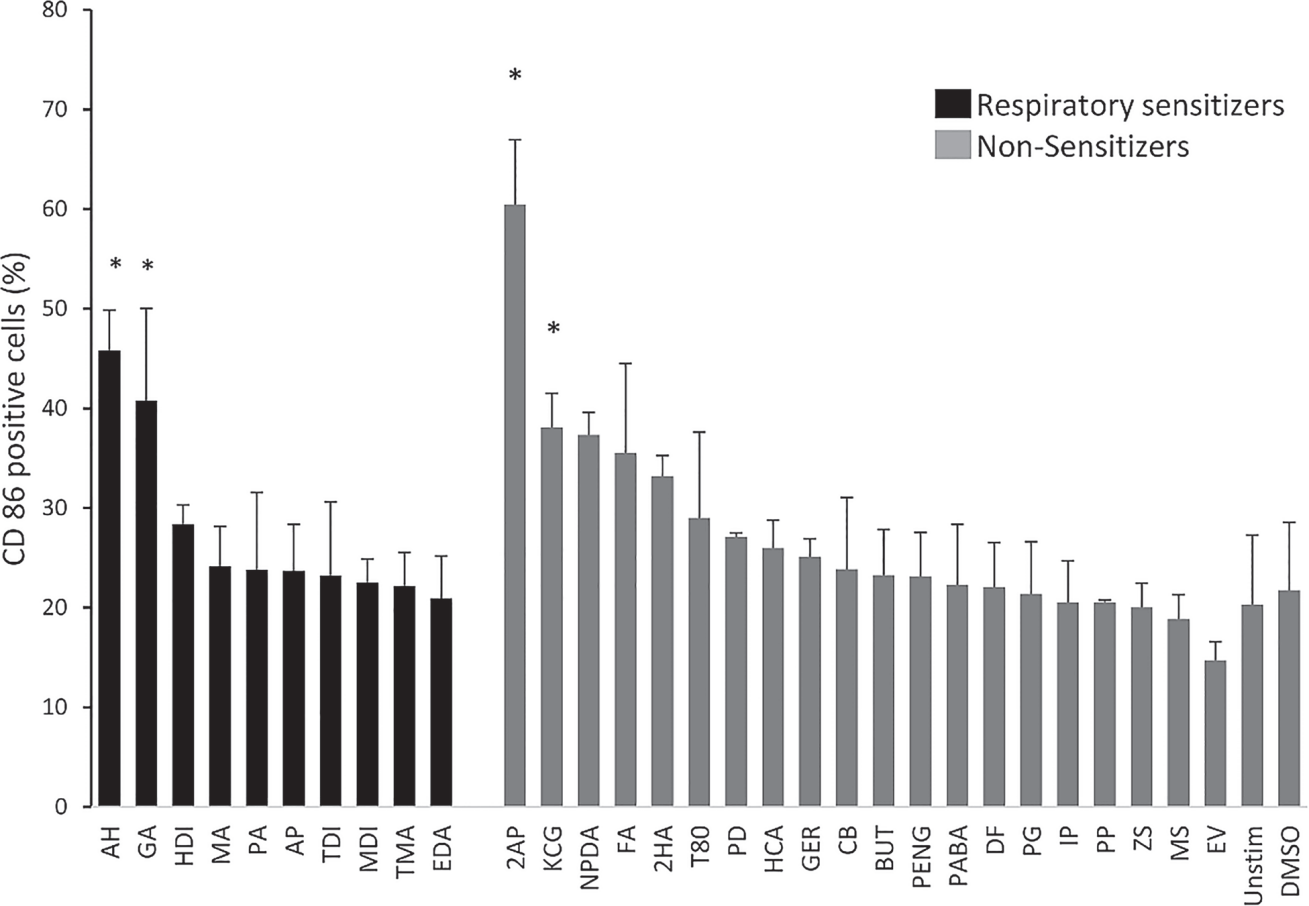
CD86 expression of MUTZ-3 cells following chemical stimulations. Data shown is an average of chemical stimulations, (n = 3), 4-Aminobenzoic acid, DMSO and unstimulated cells (n = 6) and potassium permanganate, 2-aminophenol, Hexylcinnamic aldehyde and 2-Hydroxyethyl acrylate (n = 2), with error bars showing standard deviation. Statistical significance was determined by student’s t-test, comparing each stimulation with its corresponding vehicle, with p < 0.05 indicated by *.

Prior to chemical challenge, cells were quality controlled by measuring the cellular expression of common myeloid and dendritic cell markers using flow cytometry. These markers included CD1a, CD14, CD34, CD54, CD80, CD86 and HLA-DR. Results correlated with previously published phenotypic profiles [41], ensuring that cells were successfully maintained in an immature state. Following chemical stimulation, using a panel of reference chemicals comprising 10 respiratory sensitizers and 20 non-respiratory sensitizers, as well as vehicle controls (Table 1), the general maturity state of the cells was again verified by measuring levels of cell surface expression of the co-stimulatory marker CD86, with results presented in Fig. 1. Chemically induced up-regulation of CD86 for each stimulation in comparison to unstimulated cells was evident in cells after a number of stimulations. However, due to large standard deviation between replicate stimulations, only regulation induced by the respiratory sensitizers ammonium hexachloroplatinate and glutaraldehyde could be confirmed as statistically significant (students t-test, p<0.05). Consequently, an assay using CD86 as a single biomarker for classification of respiratory sensitization would result in a sensitivity of only 20%. Additionally, up-regulation of CD86 was also observed in the non-respiratory sensitizing stimulations 2-aminophenol and kathon CG. Thus, we concluded that CD86 could not be used as a single biomarker in the MUTZ-3 cell line to classify respiratory chemical sensitizers. However, as many of the respiratory sensitizers have a poor solubility in cell media, and can thus not be used in sufficient concentration to induce cytotoxicity, the expression of CD86 may act as a complementary quality control to ensure bioavailability of chemicals stimulations.

**Table 1:** Concentrations and vehicles used for each reference compound during assay development.

Compound	Abbreviation	Vehicle	Max solubility (μM)	Rv90 (μM)	GARD input concentratrion (μM)
***Respiratory sensitizers***					
Ammonium hexachloroplatinate	AH	Water	35	-	35
Ammonium persulfate	AP	DMSO	-	-	500
Ethylenediamine	EDA	Water	-	-	500
Glutaraldehyde	GA	Water	-	10	10
Hexamethylen diisocyanate	HDI	DMSO	100	-	100
Maleic Anhydride	MA	DMSO	-	-	500
Methylene diphenol diisocyanate	MDI	DMSO	50	-	50
Phtalic Anhydride	PA	DMSO	200	-	200
Toluendiisocyanate	TDI	DMSO	40	-	40
Trimellitic anhydride	TMA	DMSO	150	-	150
					
***Non-Respiratory sensitizers***					
1-Butanol	BUT	DMSO	-	-	500
2-Aminophenol	2AP	DMSO	-	100	100
2-Hydroxyethyl acrylate	2HA	Water	-	100	100
2-nitro-1,4-Phenylenediamine	NPDA	DMSO	-	300	300
4-Aminobenzoic acid	PABA	DMSO	-	-	500
Chlorobenzene	CB	DMSO	98	-	98
Dimethyl formamide	DF	Water	-	-	500
Ethyl vanillin	EV	DMSO	-	-	500
Formaldehyde	FA	Water	-	80	80
Geraniol	GER	DMSO	-	-	500
Hexylcinnamic aldehyde	HCA	DMSO	32.34	-	32.34
Isopropanol	IP	Water	-	-	500
Kathon CG*	KCG	Water	-	0.0035%	0.0035%
Methyl salicylate	MS	DMSO	-	-	500
Penicillin G	PEN G	Water	-	-	500
Propylene glycol	PG	Water	-	-	500
Potassium Dichromate	PD	Water	51.02	1.5	1.5
Potassium permanganate	PP	Water	38	-	38
Tween 80	T80	DMSO	-	-	500
Zinc sulphate	ZS	Water	126	-	126

^*^The chemical Kathon CG is a mixture of the two compounds MC and MCI. The concentration of the mixture is given in %.

### Identification of a predictive genomic biomarker signature by transcriptional profiling of chemically stimulated MUTZ-3

Chemically induced changes in the MUTZ-3 cells were investigated on transcriptional-wide basis in order to identify the most discriminatory transcripts between respiratory sensitizers and non-respiratory sensitizers. Following 24 h of cellular stimulation with a panel of reference chemicals, mRNA was collected for transcriptional profiling. The stimulations included 10 different respiratory sensitizers, 20 non-respiratory sensitizers (negative controls) and vehicle controls (DMSO, distilled water). All stimulations were performed in biological triplicates except for 4-aminobenzoic acid, which was analyzed in 6 replicates due to internal controls, and potassium permanganate, which was analyzed in only 2 replicates due to a faulty array. In addition, vehicle controls (DMSO and distilled water) were analyzed in 6 replicates each. Quality control of samples revealed that one of the replicate stimulations of ammonium persulfate was a significant outlier and had to be removed in order not to interfere with biomarker discovery. Summarized, the data set ready for analysis consisted of 103 arrays, each with measurements of 29,141 transcripts.

**Fig 2:**
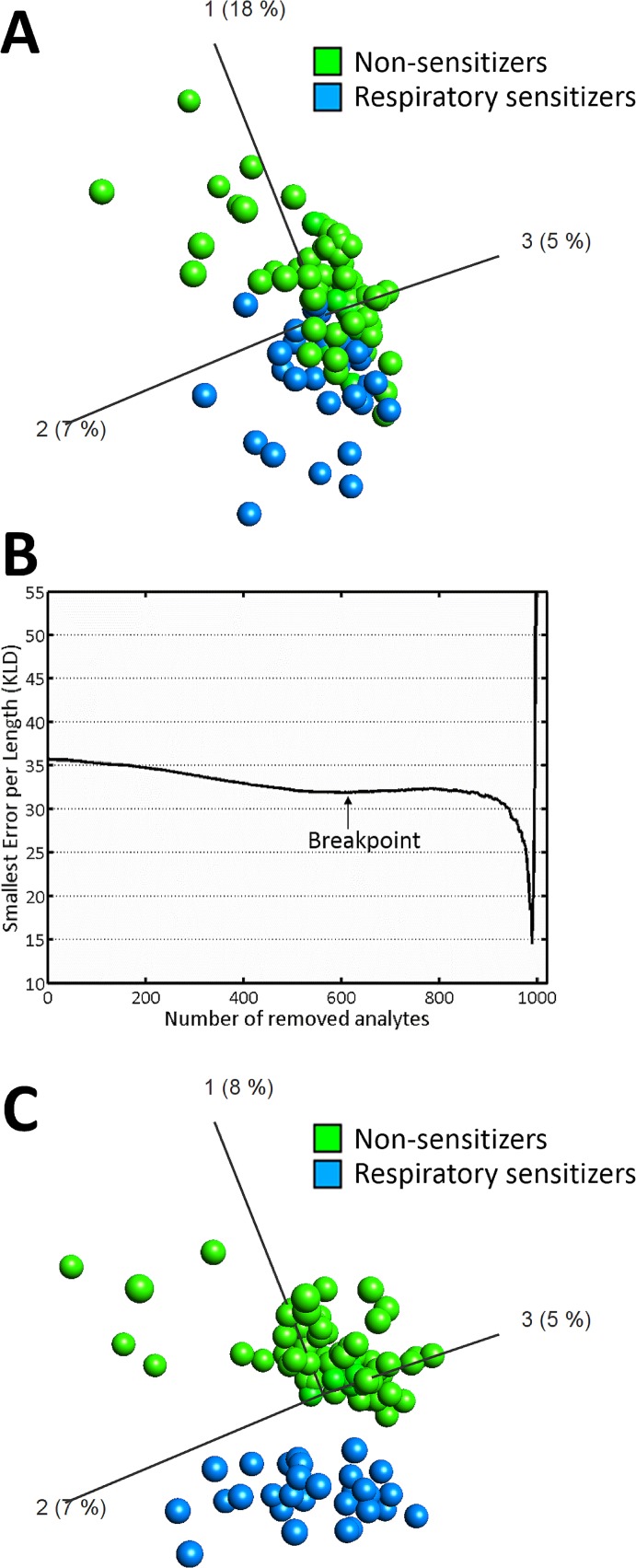
Establishment of a predictive biomarker signature for prediction of respiratory sensitization. (A) Unsupervised learning was used to construct the representation of the dataset. The method was visualized using PCA based on 999 transcripts identified by one-way ANOVA p-value filtering between respiratory sensitizers (blue, n = 29) and non-respiratory sensitizers (green, n = 74). (B) The 999 transcripts identified by p-value filtering were used as input into an algorithm for backward elimination. A breakpoint in Kullback-Leibler divergence was observed after removal of 610 transcripts. (C) The remaining 389 transcripts were used as input variables into a PCA. As illustrated in the figure, a complete seperation between respiratory sensitizers and non-respiratory sensitizers was achieved in the training data.

Gene expression data was imported into Qlucore Omics Explorer and visualized using principal component analysis (PCA). We applied a tiered approach for feature selection, combining a filtering method in order to reduce the noise in the dataset and select predictors based on intrinsic properties, and a wrapper method based on Backward Elimination in order to reduce the number of genes in the signature by taking into account how each individual predictor performs collectively with the entire signature. The filtering method was based entirely on p-values, as determined by one-way ANOVA analysis, comparing respiratory sensitizers and non-respiratory sensitizers. The wrapper method was based on repeated supervised learning. The algorithm for Backward Elimination was developed in house [52] and iteratively extracted a subset of transcripts which were subsequently evaluated by training and testing of a Support Vector machine (SVM), using a leave-one-out cross validation procedure. The least informative variables were removed, and the process was repeated until the highest performance of classification model was achieved. Due to computational limits, approximately 1000 genes is an appropriate amount of potential predictors to use as an input in the algorithm for Backward Elimination. In the present data set, this pre-selection of predictor candidates resulted in 999 genes, with a p-value of 0.024 or lower. As illustrated in Fig. 2A, these genes were collectively able to achieve a partition between respiratory sensitizers and non-respiratory sensitizers, although some overlap occurred between the two groups. Reducing the number of predictors further, by the ranking given by their p-value, did not achieve a clear separation, even though the data contained predictor candidates with p-values down to 10^–6^. The in house developed algorithm for Backward Elimination was then applied, removing the predictors (genes) that contribute the least information. A local minimum in Kullbach-Liebler Divergence (KLD) [53] was observed when 610 predictors were eliminated (Fig. 2B). The remaining 389 genes are collectively termed the “GARD Respiratory Prediction Signature” (GRPS), and their ability to differentiate between respiratory chemical sensitizers and non-respiratory sensitizers in the training dataset are illustrated in Fig. 2C. The identities of the genes are listed in S1 Table. In order to validate the method by which the biomarker signature was established, we performed a cross validation procedure where we randomly divided the samples used during biomarker discovery into a training set and a test set as described in methods. The process was iterated 20 times and the frequency of each predictor transcript among the 20 new biomarker signatures was used as a measurement of robustness. Results from this exercise were tabulated as Validation Call Frequencies (VCF) and are summarized in S1 Table.

### Visual classification of independent test compounds using GARD Respiratory Prediction Signature as an estimate of predictive performance

**Fig 3:**
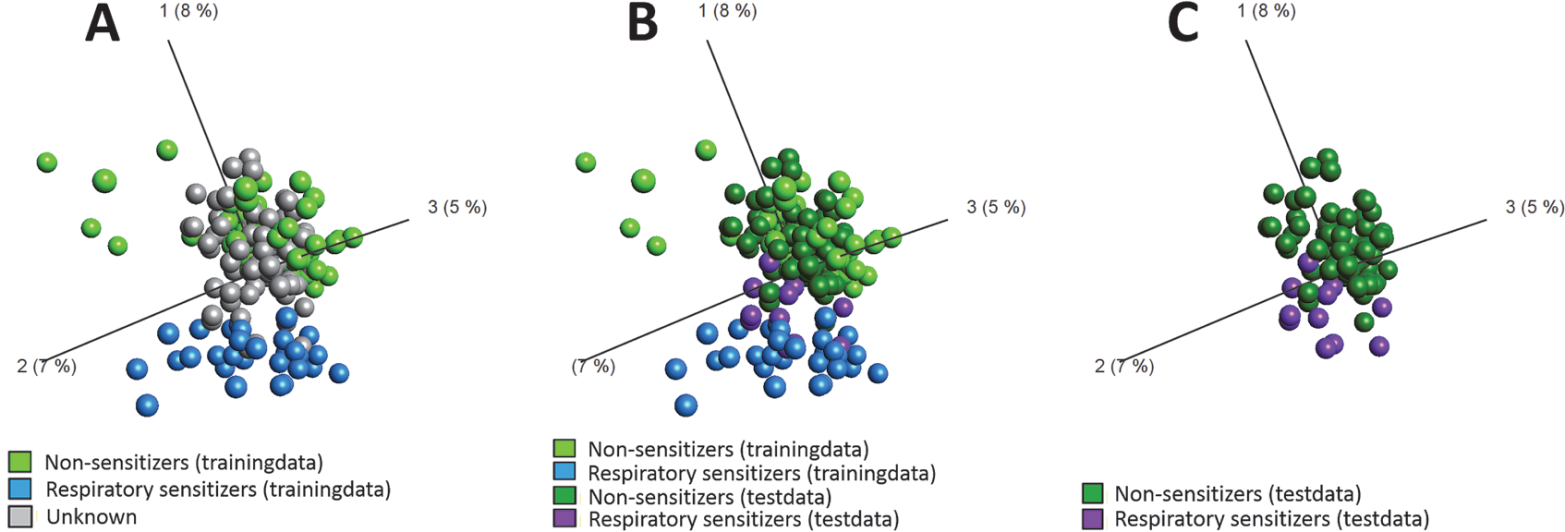
Visual classification of independent test compounds using GARD Respiratory Prediction Signature, GRPS. (A) The PCA space was constructed from the three first PCA components from the panel of reference chemicals (n = 103) used for biomarker signature identification, using the 389 genes of GRPS as input into the unsupervised representation. Each of the chemicals in the test dataset (n = 92) were plotted into the PCA space without allowing the compounds to influence PCA components. (B) Samples in the test dataset was colored according to sensitizing properties as either respiratory sensitizers (dark blue) or non-respiratory sensitizers (dark green). A separation between respiratory sensitizers and non-respiratory sensitizers can be seen along the first PCA component for both the training data and the test data. (C) The training dataset has been removed in order to obtain a clear view of the training dataset.

The predictive performance of GRPS was validated using an independent test dataset comprising both respiratory sensitizers as well as non-respiratory sensitizers in order to illustrate the relevance of the genomic biomarker signature as a predictive assay for respiratory sensitizers. The chemical compounds included in the independent test dataset are described in Table 2. Some of the compounds used during the training of the model, including 1-butanol, chlorobenzene, ethylenediamine, phtalic anhydride, and the vehicle controls (DMSO, distilled water) were included also in the independent test dataset to be used as controls. The remaining chemicals were unseen by the model prior to classification of the samples. All chemicals in the independent test dataset were based on additional stimulations, separated in time from the stimulations comprising the training dataset. Therefore, both the unseen compounds, as well as the compounds seen by the model during the identification of the GRPS could be classified without the risk of over fitting the model. Following 24 h of cellular stimulation, mRNA was collected, converted to cDNA and hybridized to the microarrays. The stimulations included 6 respiratory sensitizers, 19 non-respiratory sensitizers and vehicle controls (DMSO, distilled water). The non-respiratory sensitizing stimulations were reused from a previous set of experiments [41] and performed in biological triplicates. Stimulations performed with the respiratory sensitizers, together with the non-respiratory sensitizer 1-butanol, comprised a novel round of stimulations, and were performed in biological duplicates. The chemical chlorobenzene was included in both sets due to internal controls, hence comprised a total of 5 stimulations. In addition, vehicle controls DMSO and distilled water were analyzed in 13 and 9 replicates, respectively. In summary, the independent test dataset comprised 92 arrays. The process of performing visualized classifications of unknown samples is sequentially illustrated in Fig. 3, using the test dataset to highlight the methodology. In an initial step, the training data set, i.e. the panel of reference chemicals used to identify the predictive GRPS genomic biomarker signature, was first used to generate the PCA space, using the 389 genes in the GRPS as variable input. The PCA was then frozen in space, and each of the compounds in the test dataset was plotted into this space without allowing them to influence the PCA components (Fig. 3A). As demonstrated in Fig. 3B, upon identification of true identities of the samples in the test dataset, a clear separation between respiratory sensitizers and non-respiratory sensitizers can be seen along the first PCA component for both the training data and the test data, indicating that similarities and differences in structure of gene expression in the GPRS between respiratory sensitizers and non-respiratory sensitizers was present also in the test dataset. Fig. 3C illustrates the test dataset plotted into the frozen PCA space generated by the training dataset, but where the training dataset have been removed in order to facilitate interpretation. As seen in the figure, respiratory sensitizers and non-respiratory sensitizers appears to be separated by a hyper plane generated by the 2^nd^ and 3^rd^ principal components, indicating that the GRPS clearly contain information relevant for achieving discrimination between respiratory sensitizers and non-respiratory sensitizers in the previously unseen samples in the independent test dataset.

**Table 2:** Chemicals included in the independent dataset used for validation of GRPS.

Compound	Abbreviation	Vehicle	Max solubility (μM)	Rv90 (μM)	GARD input concentratrion (μM)
***Respiratory sensitizers***					
Chloramine T	CH-T	Water	-	-	500
Ethylenediamine	EDA	Water	-	-	500
Isophorone diisocyanate	IPDI	DMSO	25	-	25
Phtalic Anhydride	PA	DMSO	200	-	200
Piperazine	PPZ	Water	-	-	500
Reactive Orange	RO	Water	-	100	100
					
***Non-respiratory sensitizers***					
1-Butanol	BUT	DMSO	-	-	500
2,4-dinitrochlorobenzene	DNCB	DMSO	-	4	4
2-mercaptobenzothiazole	MBT	DMSO	250	-	250
Benzaldehyde	BA	DMSO	250	-	250
Chlorobenzene	CB	DMSO	98	-	98
Cinnamyl alcohol	CALC	DMSO	500	-	500
Diethyl phthalate	DP	DMSO	50	-	50
Eugenol	EU	DMSO	649	300	300
Glycerol	GLY	Water	-	-	500
Glyoxal	GO	Water	-	300	300
Isoeugenol	IEU	DMSO	641	300	300
Lactic acid	LA	Water	-	-	500
Octanoic acid	OA	DMSO	504	-	500
Phenol	PHE	Water	-	-	500
p-hydroxybenzoic acid	HBA	DMSO	250	-	250
p-phenylenediamine	PPD	DMSO	566	75	75
Resorcinol	RC	Water	-	-	500
Salicylic acid	SA	DMSO	-	-	500
Sodium dodecyl sulphate	SDS	Water	-	200	200

### SVM classifications and predictive performance of GRPS

**Fig 4:**
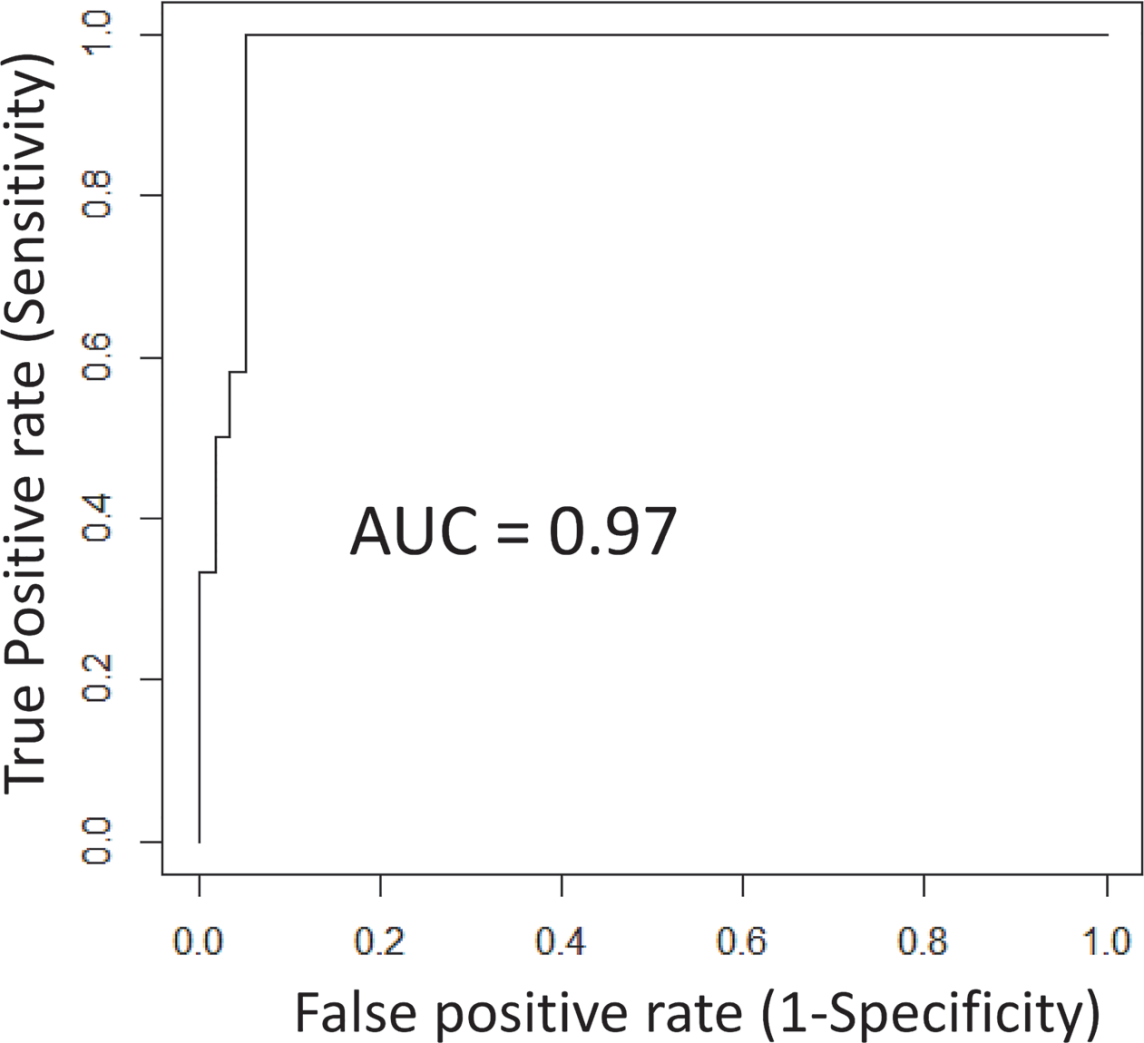
Support Vector Machine (SVM) classifications of the test dataset. The predictor performance of GRPS was validated using SVM for supervised machine learning. The SVM algorithm was inductively learned by experience to the compounds in the training dataset (n = 103) and subsequently applied to predict each individual sample in the test dataset (n = 70, vehicle controls excluded). The predictive performance was evaluated by ROC curve analysis and estimated to an Area Under the Curve (AUC) of 0.97.

**Fig 5:**
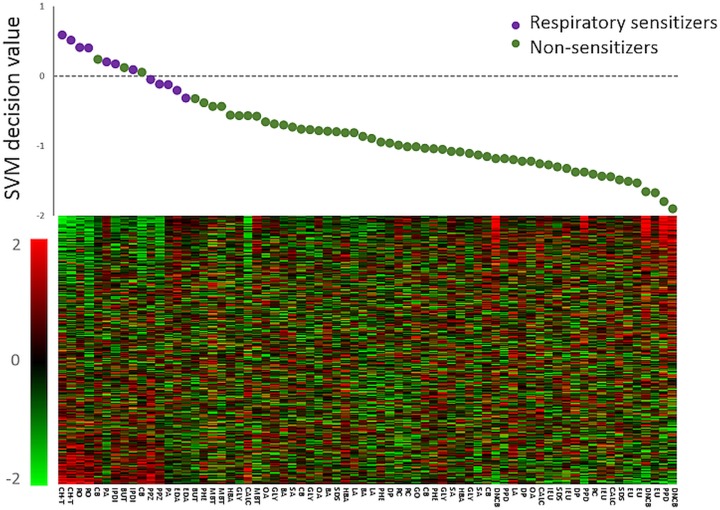
Classification and gene expression of respiratory sensitizers in the independent test dataset. The SVM algorithm was once again trained on the samples in the training dataset (n = 103) and subsequently applied in order to classify samples in the independent test dataset (n = 70, vehicle controls were excluded). SVM decision values for each individual sample in the independent test dataset were plotted in decreasing order and colored according to sensitizing capacity (Respiratory sensitizers = purple, non-respiratory sensitizers = dark green). The dotted line in the scatterplot represents the threshold level for classifications as respiratory sensitizers (SVM decision value > 0) or non-respiratory sensitizers (SVM decision value < 0). Relative expression of transcripts within GRPS is shown in the heat map.

In a consecutive step to classify the samples in the independent test dataset, the visual classifications were challenged with a binary classification model, using an SVM for supervised machine learning. The SVM was trained on the training data set to recognize differences in gene expression structure between respiratory sensitizers and non-respiratory sensitizers within the GRPS. The trained SVM model was applied to classify each sample in the independent test data set, on the level of each individual replicate, as either a respiratory sensitizer or a non-respiratory sensitizer. The output from the SVM, the SVM decision values, were compared to true identities of samples in the test dataset, and the performance of the predictor was evaluated using ROC AUC analysis with results illustrated in Fig. 4. SVM classifications were based on linear kernels with an unbiased maximum-margin hyperplane separating the two groups, hence threshold for classifications as respiratory sensitizers corresponded to a SVM decision value > 0. As shown in the figure, predictor performance of the GRPS on the level of individual stimulations was estimated to an area under the ROC curve of 0.97. Decision values obtained from the SVM classifications for each chemical compound in the test dataset are presented in Table 3 (n = 70, vehicle controls excluded). Data presented in this table is further summarized in Fig. 5. In the figure, samples are sorted in a descending scale from highest to lowest SVM value, and the SVM decision value for each compound in the test dataset are correlated to the individual expression profile of the 389 transcripts in the GRPS. In order to facilitate interpretation, samples in the figure were colored according to sensitizing capacity (Respiratory sensitizers = purple, non-respiratory sensitizers = dark green) and the threshold value for classification is illustrated by the dotted line. As illustrated in the figure, although some overlap was observed, the majority of the respiratory sensitizers was assigned SVM decision values that were higher than corresponding values assigned to non-respiratory sensitizers, which also correlated with differences in the expression profiles between the majorities of compounds within each group. For decision making and classification on sensitizing capacity for each chemical, we chose to use the cut-off stating that any given sample in the test dataset should be classified as a respiratory sensitizer if any of the replicate stimulations has an SVM decision value > 0, as determined in previously published protocols [42]. Based on this criterion, the accuracy, sensitivity and specificity of the GRPS was estimated using cooper statistics to 84%, 67%, and 89%, respectively.

**Table 3:** Results from SVM classifications of the independent test dataset.

Treatment	SVM decision value					Classification^1^
	**1**	**2**	**3**	**4**	**5**	**Pos if 1 sample > 0**
***Respiratory sensitizers***						
Chloramine T	0.52	0.59				Sensitizer
Ethylenediamine	−0.32	−0.20				Non-sensitizer
Isophorone diisocyanate	0.10	0.17				Sensitizer
Phtalic Anhydride	0.20	−0.12				Sensitizer
Piperazine	−0.05	−0.12				Non-sensitizer
Reactive Orange	0.41	0.41				sensitizer
						
***Non-respiratory sensitizers***						
1-Butanol	−0.32	0.12				Sensitizer
2,4-dinitrochlorobenzene	−1.66	−1.18	−1.90			Non-sensitizer
2-mercaptobenzothiazole	−0.44	−0.43	−0.57			Non-sensitizer
Benzaldehyde	−0.79	−0.87	−0.70			Non-sensitizer
Chlorobenzene	−1.03	−0.76	−1.15	0.24	0.06	Sensitizer
Cinnamyl alcohol	−0.57	−1.44	−1.26			Non-sensitizer
Diethyl phthalate	−1.37	−0.96	−1.22			Non-sensitizer
Eugenol	−1.67	−1.53	−1.51			Non-sensitizer
Glycerol	−1.05	−1.11	−0.77			Non-sensitizer
Glyoxal	−1.02	−0.69	−0.56			Non-sensitizer
Isoeugenol	−1.44	−1.27	−1.32			Non-sensitizer
Lactic acid	−1.20	−0.81	−0.89			Non-sensitizer
Octanoic acid	−0.65	−0.79	−1.22			Non-sensitizer
Phenol	−1.04	−0.38	−0.95			Non-sensitizer
p-hydroxybenzoic acid	−0.81	−0.56	−1.09			Non-sensitizer
p-phenylenediamine	−1.38	−1.19	−1.80			Non-sensitizer
Resorcinol	−1.01	−0.99	−1.40			Non-sensitizer
Salicylic acid	−0.73	−1.08	−1.13			Non-sensitizer
Sodium dodecyl sulphate	−1.49	−0.80	−1.30			Non-sensitizer

^1^Classification on sensitizing properties for each chemical compound was based on the rule stating that any given sample in the test dataset should be classified as a respiratory sensitizer if any of replicate stimulations have a SVM decision value > 0.

### Canonical Pathways associated with respiratory sensitizers and GARD prediction signature

Aiming to investigate the biologic response initiated by respiratory chemical sensitizers in MUTZ-3 cells, the data was analyzed using functional enrichment analysis in Metacore. The top 999 genes, selected with p-value filtering, were used as input into Metacore. Of the 999 genes, Metacore was able to map 948 to unique IDs. Significantly regulated pathways (p < 0.01) are listed in Table 4, ranked by—log (p-Value) and sorted in order of statistical significance. Genes present in GRPS are indicated in bold. A clear majority of these identified and significantly regulated pathways are mainly driven by a limited set of molecules. The most highly populated pathways included oxidative phosphorylation (26 molecules) and Ubiquinone metabolism (19 molecules), showing that cellular events such as oxidation-reduction processes and the respiratory electron transport chain function is highly affected by the studied chemicals. In addition, several of the less significantly regulated pathways, including Inhibitory PD-1 signaling in T cells, Antigen presentation by MHC class I and MHC class II, Generation of memory CD4+ T cells, IL-33 signaling pathway are relevant from an immunological point of view. Of note, central for many of these pathways is the bridge between innate and adaptive immunity, and the engagement of innate immune responses initiated by recognition of foreign substances, leading to dendritic cell maturation and activation of specific T-cell responses. Key aspects of this process is well monitored and significantly regulated in the MUTZ-3 cell line, including upregulation of antigen presentation-associated molecules, such as MHC class I and MHC class II complex, upregulation of co-stimulatory molecules, such as CD80 and CD86, and cross-talk with key players such as T-cells through initiation and coordination of pathways responsible for driving the immune response. Of note, activated pathways are only to a very limiting extent overlapping with pathways activated by skin sensitizers in MUTZ-3 [54] (Granzyme B and Granzyme A signaling), indicating that respiratory sensitizers and skin sensitizers are involved in engagement of different signaling pathways.

**Table 4:** Canonical pathways associated with the top 999 predictors able to separate respiratory chemical sensitizers from non-respiratory sensitizers.

*Canonical Pathway*	*-log(p-value)*	*Regulated molecules* ^*1*^
Oxidative phosphorylation	*17*.*62*	ATP5E, ATP5I, ATPK, COX Vb, COX VIIa-2, NDUFA1, NDUFA13, NDUFA2, NDUFA3, NDUFA6, NDUFA7, NDUFA9, NDUFAB1, NDUFB10,NDUFB4, NDUFB6, NDUFB8, NDUFB9, NDUFC1, NDUFS4, NDUFS5, NDUFS6, NDUFS8, NDUFV2, UQCR10, UQCRQPC
Ubiquinone metabolism	13.29	NDUFA1, NDUFA13, NDUFA2, NDUFA3, NDUFA6, NDUFA7, NDUFA9, NDUFAB1, NDUFB10, NDUFB4, NDUFB6, NDUFB8, NDUFB9, NDUFC1, NDUFS4, NDUFS5, NDUFS6, NDUFS8, NDUFV2
Granzyme B signaling	4.72	Bid, Caspase-2, Lamin A/C,LAMP2,**Smac/Diablo**, tBid, Tubulin alpha
FAS signaling cascades	3.79	Bid, c-FLIP (S), Caspase-2, DAXX, Lamin A/C, **Smac/Diablo**, tBid
Cytoplasmic/mitochondrial transport of proapoptotic proteins Bid, Bmf and Bim	3.57	Bid, DAXX, DLC1 (Dynein LC8a), DLC2 (Dynein LC8b), **Smac/Diablo**, tBid
Inhibitory PD-1 signaling in T cells	3.27	**BCL2L1**, **CD8**, **CD8 alpha**, CD80, CD86, MHC class II, PTEN
HSP60 and HSP70/ TLR signaling pathway	3.22	CD80, CD86, MD-2, MEK1/2, MHC class II, MyD88, Ubiquitin
Astrocyte differentiation from adult stem cells	3.17	**HES1**, ID1, ID2, ID3, MEK1/2, **SOX1**
Apoptotic TNF-family pathways	3.06	Apo-2L(TNFSF10), **BCL2L1**, Bid, Caspase-2, **Smac/Diablo**, tBid
TNFR1 signaling pathway	3.00	Bid, c-FLIP (S), Caspase-2, jBid, **Smac/Diablo**, tBid
Role of Nek in cell cycle regulation	2.80	**Histone H1**, Ran, Tubulin (in microtubules), Tubulin alpha, Tubulin beta
*ATP/ITP metabolism*	2.70	5'-NTC, ADSL, APRT, POLR2G, POLR2J, PPAP, RPB10, RPB6, RPB8, RRP41
Generation of memory CD4+ T cells	2.51	**BCL2L1**, CD80, CD86, **IL7RA,** MHC class II
Dynein-dynactin motor complex in axonal transport in neurons	2.48	DYNLL, DYNLT, Tctex-1, **TMEM108**, Tubulin (in microtubules), Ubiquitin
Antigen presentation by MHC class II	2.44	HLA-DM, HLA-DRA1, MHC class II
IL-33 signaling pathway	2.36	Histone H2A, Histone H2B, MEK1/2, MyD88, **ST2L**, Ubiquitin
Insulin regulation of translation	2.27	eEF2, eIF4A, **eIF4B**, eIF4G1/3, MEK1(MAP2K1)
TNF-alpha-induced Caspase-8 signaling	2.23	Bid, c-FLIP (S), Caspase-2, PP2A regulatory, tBid
Antigen presentation by MHC class I	2.18	**CD8,CD8 alpha**, PSMB5, **PSME3**
Main pathways of Schwann cells transformation in neurofibromatosis type 1	2.18	**BCL2L1, Calmodulin,** MEK1(MAP2K1), MEK1/2, Neuregulin 1,PTEN
Granzyme A signaling	2.08	**Histone H1**, Histone H2B, Lamin A/C, LAMP2
G-CSF-induced myeloid differentiation	2.08	G-CSF receptor, MEK1/2, Myeloblastin, PERM
Substance P mediated membrane blebbing	2.07	MRLC, Tubulin (in microtubules), Tubulin alpha
Role of IAP-proteins in apoptosis	2.03	Bid, **Smac/Diablo**, tBid, Ubiquitin

^1^Molecules indicated in bold are present in GRPS.

## Discussion

A variety of chemicals are able of inducing allergic hypersensitivity reactions in both skin and respiratory tract, eventually giving rise to clinical symptoms of Allergic Contact Dermatitis (ACD) or Occupation Asthma (OA). Although the numbers of chemicals able of inducing respiratory sensitization are far fewer in comparison to those causing skin sensitization, identification and hazard classification of respiratory chemical sensitizers remains an area of great importance due to the severe impact on health and quality of life associated with acquired OA. Development of reliable assays that accurately identifies respiratory sensitizers as well as distinguishing those from skin sensitizers have proven challenging, and to date, no validated method exists. In previous studies, we described the development and application of the Genomic Allergen Rapid Detection (GARD) assay as an *in vitro* alternative to animal testing for identification and risk assessment of skin sensitizing chemicals. In the GARD assay, unknown test chemicals are classified based on readout from a pre-determined genomic biomarker signature, measured by genome-wide transcriptional profiling. Utilizing the great versatility that comes with analyzing the complete transcriptome, we hypothesized that the applicability domain of GARD could be broadened to also cover hazard classification of respiratory sensitizers through the identification of an alternative genomic biomarker signature.

In the current study, we present a further development of GARD, allowing for classification of respiratory sensitizing chemicals, using a different biomarker signature termed the GARD Respiratory Prediction Signature (GRPS). The intended use of the defined GRPS will thus be in a novel combined *in vitro* assay, in which MUTZ-3 cells are stimulated with the unknown compounds to be classified. Of note, using the two distinct biomarker signatures, the compound can be classified as either a skin sensitizer, a respiratory sensitizer or a non-sensitizer. Chemicals that are able to induce both respiratory and skin sensitization will also be specifically classified as such.

The GRPS was identified, using a set of reference chemicals known to be either respiratory sensitizers or non-respiratory sensitizers. Differentially regulated genes in these two groups were then identified by an ANOVA p-value filtering and further optimized, using an in house developed wrapper algorithm for backward elimination. We suggest that the 389 genes in the GRPS can function as a genomic biomarker signature to discriminate between respiratory sensitizing chemicals and non-respiratory sensitizing chemicals. Assessment of the predictive performance of GRPS is an important first step in order to establish the reliability of the genomic biomarker signature for identification of respiratory sensitizers. In this study, we used a Support Vector Machine (SVM) algorithm for supervised machine learning. We trained the model to recognize structures and similarities in gene expression data in the identified GRPS genomic biomarker signature, and challenged the model with an independent test set comprising chemicals previously unseen by the model. Subsequently, we used the model to binary classify the unseen chemical compounds as either respiratory sensitizers or non-respiratory sensitizers. Performing this exercise, we demonstrated the potential of GRPS to achieve accurate predictions. The predictive performance of GRPS was estimated, using ROC AUC analysis and cooper statistics, achieving an area under the ROC curve of 0.97 and sensitivity, specificity and accuracy of 67%, 89% and 84%, respectively. This is the highest reported accuracy for hazard classification of chemicals inducing respiratory sensitization.

To date, we can only speculate on possible explanations to why the GRPS does not reach the same high sensitivity in predictions as the GPS for skin sensitizers [46], although a similar methodology for feature selection was used. It could partly be due to the smaller number of reference compounds used during assay development of GRPS in comparison to GPS, but another possible explanation could perhaps be found on the molecular level, i.e. that skin sensitizers are more potent regulators of gene expression in MUTZ-3 cells. Irrespectively, the use of whole genome arrays as readout for classifications still makes the GRPS highly flexible. As more samples are analyzed, additional information can easily be implemented into the assay to improve sensitivity, specificity and accuracy and to fine-tune the methodology to reflect the diversity of available chemical compounds.

To further explore the biological effects of sensitizing chemicals on MUTZ-3, an enrichment analysis was performed. In order to achieve sufficient significance in the data, the top 999 genes from p-value filtering were used as input in the Metacore software, rather than the top 389 genes of the GRPS. Without doubt, the most highly populated pathways initiated by the respiratory sensitizers were involved in cellular events such as oxidation-reduction processes and respiratory electron transport chain (see Table 4). These molecules were among the top genes from the p-value filtering procedure, and not present in the GRPS signature. In this respect, it is important to distinguish between functionality, in this case aiming at describing the biological relevance of the transcripts, and the GRPS prediction profile, aiming at performing accurate classification of independent samples. Several of the molecules involved in the oxidative phosphorylation and ubiquinone metabolism pathways are subunits of protein complexes, and thus spatially and temporary linked. The Backward Elimination procedure applied during feature selection in this study is based on orthogonal selection of variables, thus, features that did not contribute to orthogonal information were removed during this process. Therefore, it is not surprising, but rather expected, that some of the significantly regulated pathways did not contain, or only contained a few transcripts from the GRPS signature as e.g. subunits in a molecular complex will likely have a similar expression pattern. Based on several of the less activated pathways, the biological response in MUTZ-3 to chemical respiratory allergens involves also regulation of innate immune response signaling pathways that ultimately results in cell maturation, leading to enhanced antigen presentation and interaction with other immune cells. Furthermore, novel findings of usage of signaling pathways that has previously been associated with respiratory sensitization to protein allergens will shed light on the biological process leading to sensitization of the respiratory tract in response to chemical allergens. Thus, the GRPS is indeed relevant in an immunologically mechanistic perspective, and provides measurement of transcripts that monitor the biologic events leading to respiratory sensitization.

Further, results from enrichment analysis along with the results presented for the GARD assay, demonstrates that MUTZ-3 is a suitable model for prediction of both skin- and respiratory sensitizers. Despite some similarities in immunobiological mechanisms, important mechanistic differences exist between skin- and respiratory sensitization. Skin sensitization is primary associated with induction of Th1 cells, promoting a cytotoxic CD8+ T-cell response and secretion of IL-2 and interferon (IFN)-γ, while respiratory sensitization generally involve CD4+ Th2 cells and are characterized by high levels of IL-4, IL-10 and IL-13. Although respiratory sensitization to protein antigens are driven by the production of specific IgE antibody, it is still unclear what role the IgE antibody has during the development of respiratory allergy to chemical allergens, and whether there are mechanisms through which respiratory sensitization can be achieved that are independent of IgE antibody production [55,56]. It has been suggested that it may be sufficient with an induced Th2 response, without the need of IgE, to support the development of respiratory sensitization [57]. Although clear differences in T-cell responses, activation of dendritic cells (DCs) is common for both skin- and respiratory sensitization. Consequently, DCs are natural targets for assay development in terms of both skin and respiratory sensitization due to their physiological roles during initiation, modulation and polarization of immune responses in response to xenobiotic compounds. The MUTZ-3 cell line resembles primary dendritic cells (DCs) in terms of expression profile and ability to activate specific T-cell responses [45]. In comparison to primary DCs, MUTZ-3 are easy to grow using standardized protocols and provides a sustainable source of cells, offering an opportunity to scale up the assay to a high-throughput format.

In the context of developing an assay for both skin- and respiratory sensitization, it is important to acknowledge the formal semantics behind the nomenclature. Analogous to others [24,58–60], we use the terminology to indicate the local site of the immunological response and not the route of initial exposure in this study. For example, it has been shown that sensitization of the respiratory tract can arise also after dermal exposure [61–63] to relevant chemicals. In general, a certain chemical compound selectively sensitizes either the skin or the respiratory tract. However, during certain circumstances and in immunologically susceptible individuals, some chemicals have been shown to give rise to both type of sensitization. For example, the chemical triglycidylisocyanurate (TGIC) has been shown to cause both OA and ACD [64]. In the context of this study, the two chemicals ethylenediamine [65] and piperazine [66] have been implicated to give rise to both responses. These two compounds were predicted as false negatives in the GRPS during the validation using the independent dataset. Hence, it seems that the GRPS, in present form, is not able to accurately predict chemicals that are associated with both responses. This may partly explain the somewhat low sensitivity achieved in this study. Of note, both compounds are accurately predicted as skin sensitizers in the GARD assay.

Finally, the approach of the GARD assay has several advantages, in comparison to other alternative methods. Using our data driven methodology, we were able to circumvent problems associated with the current shortage in knowledge regarding the exact mechanisms by how respiratory sensitizers provoke immunological responses in susceptible individuals. Secondly, the large amount of information obtained by the transcriptome-wide approach provides an additional opportunity to elucidate molecular mechanisms, such as specific signaling or metabolic pathways involved in the process of respiratory sensitization.

The major aim of this study was to develop an *in vitro* method in accordance with the three Rs principle on reduction, refinement and replacement of animal experiments for prediction of respiratory sensitization. Having trained a model with a set of reference chemicals, we present a tool to determine whether an unknown chemical is likely to behave as a non-respiratory sensitizer or a respiratory sensitizer. In the future, as the gaps in the current knowledge of how chemicals cause sensitization in the respiratory tract continues to be filled in, a consensus similar to the formulation of Adverse Outcome Pathways (AOP) for skin sensitization [67] may be a reality also for testing of respiratory sensitizers. The GRPS will then be an appealing part of an Integrated Testing Strategy (ITS), useful for assessment of DC maturation.

In conclusion, this study presents a predictive biomarker signature for classification of respiratory chemical sensitizers in MUTZ-3 cells that complement the previously described GARD assay for assessment of skin sensitizers. The ability to test for two different endpoints in the same sample provides an attractive and hitherto unique assay for safety assessment of chemicals in an *in vitro* testing strategy that comply with the three R principle on reduction, refinement and replacement of animal experiments.

## Methods

### Chemicals

A panel of 32 reference chemicals comprising a selection of 10 well characterized respiratory sensitizers and 22 non-respiratory sensitizers, collectively termed the training dataset, were used for cell stimulations in order to establish the predictive genomic biomarker signature. The respiratory sensitizers were ammonium hexachloroplatinate, ammonium persulfate, ethylenediamine, glutaraldehyde, hexamethylene diisocyanate, maleic anhydride, methylene diphenyl diisocyanate, phtalic anhydride, toluene diisocyanate and trimellitic anhydride. The non-respiratory sensitizers were 1-butanol, 2-aminophenol, 2-hydroxyethyl acrylate, 2-nitro-1,4-phenylenediamine, 4-aminobenzoic acid, chlorobenzene, dimethyl formamide, ethyl vanillin, formaldehyde, geraniol, hexylcinnamic aldehyde, isopropanol, Kathon CG, methyl salicylate, penicillin G, potassium dichromate, potassium permanganate, propylene glycol, Tween 80, zinc sulfate and the vehicle controls dimethyl sulfoxide and water. Additionally, a panel of 25 chemicals, including 6 respiratory sensitizers and 19 non-respiratory sensitizers, collectively termed the independent test dataset, were used for cell stimulations in order to form an independent testset for validation of the identified predictive genomic biomarker signature. The independent training dataset comprised both control chemicals, included during the training of the model, as well as chemicals previously unseen during training of the model. The respiratory sensitizers were chloramine T, ethylenediamine, Isophorone diisocyanate, phtalic anhydride, piperazine and reactive orange. The non-respiratory sensitizers were 1-butanol, 2,4-dinitrochlorobenzene, 2-mercaptobenzothiazole, benzaldehyde, chlorobenzene, cinnamyl alcohol, diethyl phthalate, eugenol, glycerol, glyoxal, isoeugenol, lactic acid, octanoic acid, phenol, p-hydroxybenzoic acid, p-phenylendiamine, resorcinol, salicylic acid and sodium dodecyl sulfate. All chemicals were purchased from Sigma-Aldrich (St. Louis, MO, USA). Chemicals were dissolved and diluted into GARD input concentration in either water or DMSO prior to stimulation of cells. For chemicals dissolved in DMSO, the in-well concentration of DMSO was 0.1%. Monitoring of chemical cytotoxicity and establishment of GARD input concentration for each chemical compound was performed as previously described [41,42]. In short, GARD input concentration was determined according to the following decision schedule: Non-toxic and freely soluble compounds were used at a concentration corresponding to 500 uM. Non-toxic and poorly soluble compounds, insoluble at 500 uM, were used at highest soluble concentration. Toxic compounds were used at a concentration yielding 90% relative viability (Rv90). The criterion that was first met determined the GARD input concentration for each compound. The GARD input concentration, sensitizing potency and solvent are presented in Table 1 for compounds used to establish the predictive genomic biomarker signature, and in Table 2 for compounds used to validate the predictive genomic biomarker signature.

### Cell cultures, phenotypic analysis, chemical exposure, cell harvest and mRNA isolation

The human acute myelomonocytic leukemia cell line MUTZ-3[68,69] was obtained from Leibniz-Institut DSMZ-Deutsche Sammlung von Mikroorganismen und Zellkulturen (DSMZ, Braunschweig, Germany). Maintenance of cells, chemical stimulation of MUTZ-3 and all subsequent isolation of mRNA and preparation of cDNA were performed as previously described [41,42]. In short, a phenotypic control of MUTZ-3 was performed using flow cytometry prior to chemical stimulation to ensure cells were in an immature state. The following FITC-conjugated mouse monoclonal antibodies (mAbs) were used: CD1a (DakoCytomation, Glostrup, Denmark), CD34, CD86, HLA-DR, IgG1 (BD Biosciences, Franklin Lakes, NJ). The following PE-conjugated mouse monoclonal antibodies were used: CD14 (DakoCytomation), CD54, CD80, IgG1 (BD Biosciences). Cell viability was determined using Propidium Iodide (BD Biosciences) staining. Samples were run on FACSCanto II instrument. Data was acquired using FACS Diva software (BD Biosciences) and analyzed using FCS Express V4 (De Novo Software, Los Angeles, CA). Gating was performed to exclude cell debris and non-viable cells based on forward- and side-scattering properties and quadrants established using isotype-controls. During chemical exposure, cells were seeded at 200.000 cells/ml in 24-well plates and exposed to chemical compound at the GARD input concentration. Stimulated cells were harvested after 24h incubation at 37^o^C, 5% CO_2_ and RNA was isolated with TRIzol reagent (Life Technologies, Carlsbad, CA) using standardized protocols provided by the manufacturer. In parallel, a control of the maturity state of the cells was performed by flow cytometric analysis of CD86. Preparation of cDNA and hybridization, washing and scanning of the Human Gene 1.0 ST Arrays (Affymetrix, Santa Clara, CA, USA) was performed, according to standardized protocols provided by the manufacturer (Affymetrix).

### Microarray data analysis and statistical methods

Gene expression data obtained from the Human Gene 1.0 ST Arrays were normalized using the Single-Channel array normalization (SCAN) algorithm [70], and potential batch effects between different set of experiments were adjusted using the ComBat [71] empirical bayes method. Normalizations and batch adjustments were performed in R statistical software [72] using the open software Bioconductor v2.14 [73] with the additional software packages SCAN.UPC [70] and sva [74]. Normalized data was imported into Qlucore Omics Explorer 3.0 (Qlucore AB, Lund, Sweden) and visualized using Principal Component Analysis (PCA) [75]. Predictors were selected from a one-way ANOVA p-value filtration, using false discovery rate (FDR) [76] to adjust for multiple hypothesis testing, comparing respiratory sensitizers and non-respiratory sensitizers. A wrapper algorithm for Backward Elimination [41,52] was applied on the top 999 predictors, to further reduce and refine the biomarker signature size. The Backward Elimination algorithm was modified to minimize the Kullback-Leibler error [53] rather than maximizing the Area Under the Receiver Operating Characteristic (AUC ROC) [77], in order to enable signature optimization in cases where the AUC ROC reaches 1.0. The selected top 389 predictors after backward elimination were collectively designated “GARD Respiratory Prediction Signature”. The script for Backwards Elimination was programmed in R, with the additional package e1071 [78]. The method by which the predictive genomic biomarker signature was established was validated using cross-validation based on Support Vector Machines (SVM) [79], based on a linear kernel, as described previously [41]. In short, the training dataset was randomly divided into a new cross-validation training dataset comprising 70% of the stimulations, and a cross-validation test dataset comprising 30% of the stimulations. In addition, care was taken to maintain the same proportion between respiratory sensitizers and non-respiratory sensitizers as in the complete training dataset. A new predictive genomic biomarker signature was identified from the cross-validation training dataset using one-way ANOVA p-value filtration as described above. The identified predictive biomarker signature was used to train a SVM based on the information in the cross-validation training dataset. SVMs were compiled in R statistical software with the additional package e1071. The SVM model was subsequently used to predict the samples of the cross-validation test dataset. The process of biomarker identification was repeated 20 times and the robustness of the feature selection process was evaluated by calculating the frequency (referred to as the Validation call frequency, of VCF) by which each individual transcript was included in the 20 training datasets. The predictive performance of the GRPS in terms of prediction of unknown samples was estimated using the independent test dataset as described in [46]. In short, a SVM was trained on the training dataset, using the GRPS as variable input. Subsequently, the SVM was then used to predict the samples in the training dataset in the same way as described for the cross-validation above, and the predictive performance of the model was evaluated using AUC ROC, determined in R statistical environment using the additional package ROCR [80]. Classification of samples as respiratory sensitizers or non-respiratory sensitizers were based on SVM decision values on replicate level. Hence, a chemical was classified as a respiratory sensitizer if any of the replicate stimulations from a certain chemical stimulation had an SVM decision value > 0. The accuracy, sensitivity and selectivity of the assay was determined using cooper statistics [81]. The biological relevance of the GRPS was explored using MetaCore (Thomson Reuters, New York, NY) by performing a functional enrichment. The top 999 predictors from a p-value filtering were used as input into the MetaCore algorithm and biological relevance was established by exploring the Canonical Pathways associated with input molecules. The array data from training dataset has been uploaded to ArrayExpress (http://www.ebi.ac.uk/arrayexpress/) with accession number E-MEXP-3773.

## Supporting Information

S1 Table GRPS prediction signature. The table shows predictor genes in GRPS, identified by one-way ANOVA p-value filtering and Backward elimination. When possible, the Ensembl transcript ID was used as gene identifier. **Legend:** ^1^Validation call frequency (%) describes the occurrence of each predictor transcript among the 20 biomarker signatures obtained by cross validation.
(DOCX)
