# Supplementary material for: Prediction of Chemical Respiratory Sensitizers Using GARD, a Novel In Vitro Assay Based on a Genomic Biomarker Signature
Source: PLoS One. 2015 Mar 11;10(3):e0118808. doi: 10.1371/journal.pone.0118808 (PMC4356558; doi:10.1371/journal.pone.0118808)
Supplement: S1 Table — The table shows predictor genes in GRPS, identified by one-way ANOVA p-value filtering and Backward elimination. When possible, the Ensembl transcript ID was used as gene identifier. Legend: 1Validation call frequency (%) describes the occurrence of each predictor transcript among the 20 biomarker signatures obtained by cross validation. (DOCX) [file pone.0118808.s001.docx]

**Table S1**. GRPS prediction signature.

| Gene ID | Ensembl Transcript ID | Affymetrix  Probe set ID | Validation call frequency^1^ |
| --- | --- | --- | --- |
| C11orf73 | ENST00000278483 | 7942932 | 100 |
| OR5B21 | ENST00000278483 | 7948330 | 100 |
| TNFRSF19 | ENST00000403372 | 7968015 | 100 |
| NOX5 /// SPESP1 | ENST00000395421 | 7984488 | 100 |
| SNORA74A | NR_002915 | 8108420 | 100 |
| SPAM1 | ENST00000340011 | 8135835 | 100 |
| --- | ENST00000364621 | 7917972 | 95 |
| AMICA1 | ENST00000356289 | 7952022 | 95 |
| HOMER3 | ENST00000392351 | 8035566 | 95 |
| CD1C | ENST00000368169 | 7906348 | 90 |
| IGHD /// IGHM | ENST00000390538 | 7981601 | 90 |
| SNRPN /// SNORD116-26 | NR_003340 | 7982000 | 90 |
| --- | ENST00000387422 | 8159963 | 90 |
| --- | ENST00000364678 | 7934896 | 85 |
| STRAP | ENST00000025399 | 7954173 | 85 |
| DIABLO | ENST00000267169 | 7967230 | 85 |
| SERPINB1 | ENST00000380739 | 8123598 | 85 |
| --- | ENST00000411349 | 8151989 | 85 |
| --- | ENST00000385497 | 7923037 | 80 |
| OR51A2 | ENST00000380371 | 7946017 | 80 |
| MRPL21 | ENST00000362034 | 7949995 | 80 |
| PPP1R14A | ENST00000301242 | 8036473 | 80 |
| DEFB127 | ENST00000382388 | 8060314 | 80 |
| --- | ENST00000387396 | 8065752 | 80 |
| C9orf130 | ENST00000375268 | 8162562 | 80 |
| CD1A | ENST00000289429 | 7906339 | 75 |
| PRO2012 | BC019830 | 7924817 | 75 |
| LOC399898 | AK128188 | 7940116 | 75 |
| --- | ENST00000387701 | 7969914 | 75 |
| WDR68 | ENST00000310827 | 8009164 | 75 |
| NEU2 | ENST00000233840 | 8049243 | 75 |
| --- | ENST00000386677 | 8072575 | 75 |
| SPARC | ENST00000231061 | 8115327 | 75 |
| --- | ENST00000390342 | 8139107 | 75 |
| RAB9A | ENST00000243325 | 8166098 | 75 |
| CRNN | ENST00000271835 | 7920178 | 70 |
| C10orf90 | ENST00000356858 | 7936996 | 70 |
| MMP12 | ENST00000326227 | 7951297 | 70 |
| ACVRL1 | ENST00000267008 | 7955562 | 70 |
| EIF4E2 | ENST00000258416 | 8049180 | 70 |
| RP11-191L9.1 | ENST00000380990 | 8076819 | 70 |
| PDCD6 /// AHRR | ENST00000264933 | 8104180 | 70 |
| ARRDC3 | ENST00000265138 | 8113073 | 70 |
| VWDE | ENST00000275358 | 8138258 | 70 |
| ZBTB34 | ENST00000319119 | 8157945 | 70 |
| ITGB1BP2 | ENST00000373829 | 8168291 | 70 |
| OR10K2 | ENST00000392265 | 7921356 | 65 |
| LPXN | ENST00000263845 | 7948332 | 65 |
| FLJ22596 | AK026249 | 7950442 | 65 |
| --- | ENST00000306515 | 8043572 | 65 |
| ACVR2A | ENST00000404590 | 8045587 | 65 |
| GGTLC2 | ENST00000215938 | 8071662 | 65 |
| --- | ENST00000385690 | 8092312 | 65 |
| --- | ENST00000386018 | 8097945 | 65 |
| C6orf201 | ENST00000360378 | 8116696 | 65 |
| --- | ENST00000385583 | 8136932 | 65 |
| --- | ENST00000385719 | 8148515 | 65 |
| GPR20 | ENST00000377741 | 8153269 | 65 |
| --- | ENST00000364357 | 8163084 | 65 |
| ZCCHC13 | ENST00000339534 | 8168420 | 65 |
| GPR64 | ENST00000356606 | 8171624 | 65 |
| CD1D | ENST00000368171 | 7906330 | 60 |
| DUSP12 | ENST00000367943 | 7906810 | 60 |
| KLHL33 | ENST00000344581 | 7977567 | 60 |
| PSMB6 | ENST00000270586 | 8003953 | 60 |
| TMEM95 | ENST00000396580 | 8004364 | 60 |
| C1QBP | ENST00000225698 | 8011850 | 60 |
| EMILIN2 | ENST00000254528 | 8019912 | 60 |
| --- | ENST00000384680 | 8051862 | 60 |
| CD8A | ENST00000352580 | 8053584 | 60 |
| C20orf152 | ENST00000349339 | 8062237 | 60 |
| KCNJ4 | ENST00000303592 | 8076072 | 60 |
| --- | ENST00000364163 | 8078310 | 60 |
| FAM19A1 | ENST00000327941 | 8080918 | 60 |
| --- | ENST00000384601 | 8081233 | 60 |
| POLR2H | ENST00000296223 | 8084488 | 60 |
| --- | AK000420 | 8110706 | 60 |
| --- | ENST00000363354 | 8120360 | 60 |
| --- | --- | 8121483 | 60 |
| EGFL6 | ENST00000361306 | 8166079 | 60 |
| POU3F4 | ENST00000373200 | 8168567 | 60 |
| PNPLA4 | ENST00000381042 | 8171229 | 60 |
| --- | ENST00000385841 | 7905629 | 55 |
| CAMK1D | ENST00000378845 | 7926223 | 55 |
| OR52A5 | ENST00000307388 | 7946023 | 55 |
| TIMM8B | ENST00000280354 | 7951679 | 55 |
| PEBP1 | ENST00000261313 | 7959070 | 55 |
| OR4F6 | ENST00000328882 | 7986530 | 55 |
| CDH15 | ENST00000289746 | 7997880 | 55 |
| TMEM199 | ENST00000292114 | 8005857 | 55 |
| ABI3 | ENST00000225941 | 8008185 | 55 |
| FLJ42842 | AK124832 | 8008540 | 55 |
| MC4R | ENST00000299766 | 8023593 | 55 |
| --- | ENST00000410673 | 8045931 | 55 |
| ISM1 | ENST00000262487 | 8061013 | 55 |
| LOC440957 | ENST00000307106 | 8080416 | 55 |
| KLB | ENST00000257408 | 8094679 | 55 |
| GM2A | ENST00000357164 | 8109344 | 55 |
| ANXA6 | ENST00000354546 | 8115234 | 55 |
| --- | ENST00000410754 | 8120979 | 55 |
| TAS2R40 | ENST00000408947 | 8136846 | 55 |
| --- | --- | 8142880 | 55 |
| RARRES2 | ENST00000223271 | 8143772 | 55 |
| SH2D4A | ENST00000265807 | 8144880 | 55 |
| PLP1 | ENST00000361621 | 8169061 | 55 |
| ATP1A2 | ENST00000392233 | 7906501 | 50 |
| CDC123 | ENST00000281141 | 7926207 | 50 |
| --- | ENST00000386800 | 7932610 | 50 |
| MAT1A | ENST00000372206 | 7934755 | 50 |
| TSGA10IP | ENST00000312452 | 7941469 | 50 |
| PRDM7 | ENST00000325921 | 8003571 | 50 |
| --- | ENST00000390847 | 8015739 | 50 |
| WDFY1 | ENST00000233055 | 8059361 | 50 |
| --- | ENST00000255183 | 8066444 | 50 |
| MRPL39 | ENST00000307301 | 8069620 | 50 |
| --- | ENST00000386327 | 8074884 | 50 |
| TIPARP | ENST00000295924 | 8083569 | 50 |
| HES1 | ENST00000232424 | 8084880 | 50 |
| --- | ENST00000363502 | 8089727 | 50 |
| PRDM9 | ENST00000253473 | 8104634 | 50 |
| --- | ENST00000390917 | 8137433 | 50 |
| KIAA1688 | ENST00000377307 | 8153876 | 50 |
| --- | ENST00000391219 | 8156759 | 50 |
| --- | ENST00000387973 | 8160782 | 50 |
| hCG_1749005 | --- | 8167640 | 50 |
| LOC100129534 | --- | 7911718 | 45 |
| SLC2A1 | ENST00000397019 | 7915472 | 45 |
| CD48 | ENST00000368046 | 7921667 | 45 |
| --- | AF116714 | 7935359 | 45 |
| EPS8L2 | ENST00000318562 | 7937443 | 45 |
| MED19 | ENST00000337672 | 7948293 | 45 |
| MGC3196 | ENST00000307366 | 7948836 | 45 |
| --- | --- | 7952733 | 45 |
| --- | ENST00000384391 | 7990031 | 45 |
| EME2 | ENST00000307394 | 7992379 | 45 |
| NETO1 | ENST00000299430 | 8023828 | 45 |
| NPHS1 | ENST00000353632 | 8036176 | 45 |
| --- | ENST00000384109 | 8047215 | 45 |
| DRD5 | ENST00000304374 | 8053725 | 45 |
| --- | ENST00000364143 | 8059799 | 45 |
| ISX | ENST00000404699 | 8072636 | 45 |
| IL17RB | ENST00000288167 | 8080562 | 45 |
| PCOLCE2 | ENST00000295992 | 8091243 | 45 |
| LRIT3 | ENST00000409621 | 8096839 | 45 |
| --- | ENST00000330110 | 8104615 | 45 |
| ZNF354C | ENST00000315475 | 8110491 | 45 |
| --- | ENST00000386444 | 8162927 | 45 |
| OR2G3 | ENST00000320002 | 7911209 | 40 |
| GLUL | ENST00000331872 | 7922689 | 40 |
| CCKBR | ENST00000334619 | 7938090 | 40 |
| OR1S2 | ENST00000302592 | 7948312 | 40 |
| DCUN1D5 | ENST00000260247 | 7951325 | 40 |
| --- | ENST00000388291 | 7951420 | 40 |
| EMG1 | ENST00000261406 | 7953594 | 40 |
| PTHLH | ENST00000395868 | 7962000 | 40 |
| PTGES3 | ENST00000262033 | 7964250 | 40 |
| --- | --- | 7967586 | 40 |
| CIDEB | ENST00000258807 | 7978272 | 40 |
| --- | ENST00000383863 | 7985918 | 40 |
| ATP10A | ENST00000356865 | 7986789 | 40 |
| MYO5C | ENST00000261839 | 7988876 | 40 |
| --- | ENST00000380078 | 7989951 | 40 |
| PLA2G10 | ENST00000261659 | 7999588 | 40 |
| VAPA | ENST00000340541 | 8020129 | 40 |
| HSPE1 | ENST00000409729 | 8047223 | 40 |
| --- | ENST00000388324 | 8096249 | 40 |
| MYO6 | ENST00000369977 | 8120783 | 40 |
| C7orf30 | ENST00000287543 | 8131860 | 40 |
| FAM71F1 | ENST00000315184 | 8135945 | 40 |
| --- | ENST00000340779 | 8139828 | 40 |
| LOC441245 | AK090474 | 8139887 | 40 |
| CRIM2 | ENST00000297801 | 8142821 | 40 |
| XKR4 | ENST00000327381 | 8146475 | 40 |
| FAM110B | ENST00000361488 | 8146533 | 40 |
| PEBP4 | ENST00000256404 | 8149725 | 40 |
| LOC644714 | BC047037 | 8161943 | 40 |
| PAPPAS | AY623011 /// AY623012 | 8163672 | 40 |
| BEX4 | ENST00000372691 | 8169009 | 40 |
| HMGB4 | ENST00000323936 | 7899905 | 35 |
| --- | BC028413 /// BC128516 | 7911676 | 35 |
| --- | ENST00000363919 | 7928750 | 35 |
| --- | ENST00000335621 | 7958942 | 35 |
| SOX1 | ENST00000330949 | 7970146 | 35 |
| CTSG | ENST00000216336 | 7978351 | 35 |
| --- | ENST00000362344 | 7982100 | 35 |
| FLJ37464 | ENST00000398354 | 7996377 | 35 |
| RAX | ENST00000334889 | 8023549 | 35 |
| IL29 | ENST00000333625 | 8028613 | 35 |
| CEACAM20 | ENST00000316962 | 8037482 | 35 |
| --- | ENST00000365557 | 8044684 | 35 |
| SEC14L3 | ENST00000403066 | 8075375 | 35 |
| C3orf52 | ENST00000264848 | 8081645 | 35 |
| FETUB | ENST00000265029 | 8084657 | 35 |
| PIGY | ENST00000273968 | 8101718 | 35 |
| CDH12 | ENST00000284308 | 8111234 | 35 |
| LGSN | ENST00000370657 | 8127380 | 35 |
| --- | ENST00000391031 | 8129067 | 35 |
| HGC6.3 | AB016902 | 8130824 | 35 |
| tcag7.873 | NM_001126493 | 8138797 | 35 |
| --- | --- | 8141421 | 35 |
| T1560 | ENST00000379496 | 8146527 | 35 |
| EXOSC4 | ENST00000316052 | 8148710 | 35 |
| TRAM1 | ENST00000262213 | 8151281 | 35 |
| --- | --- | 8159371 | 35 |
| OR13C2 | ENST00000318797 | 8162936 | 35 |
| HCCS | ENST00000321143 | 8165995 | 35 |
| PLS3 | ENST00000289290 | 8169473 | 35 |
| TMEM53 | ENST00000372244 | 7915578 | 30 |
| CD1B | ENST00000368168 | 7921346 | 30 |
| SORCS3 | ENST00000393176 | 7930341 | 30 |
| OR52E8 | ENST00000329322 | 7946111 | 30 |
| FAM160A2 | ENST00000265978 | 7946128 | 30 |
| LOC649946 | BC017930 | 7952126 | 30 |
| FAM158A | ENST00000216799 | 7978114 | 30 |
| --- | --- | 7986637 | 30 |
| MYO1E | ENST00000288235 | 7989277 | 30 |
| NUPR1 | ENST00000395641 | 8000574 | 30 |
| --- | --- | 8005433 | 30 |
| SIGLEC15 | ENST00000389474 | 8021091 | 30 |
| 2-Mar | ENST00000393944 | 8025421 | 30 |
| LOC100131554 | --- | 8041886 | 30 |
| GGTLC1 | ENST00000335694 | 8065427 | 30 |
| PSMA7 | ENST00000395567 | 8067382 | 30 |
| SLC25A18 | ENST00000399813 | 8071107 | 30 |
| C3orf14 | ENST00000232519 | 8080847 | 30 |
| CDX1 | ENST00000377812 | 8109226 | 30 |
| --- | ENST00000386433 | 8121249 | 30 |
| RRAGD | ENST00000359203 | 8128123 | 30 |
| SDK1 | ENST00000389531 | 8131205 | 30 |
| LOC168474 | NR_002789 | 8139826 | 30 |
| --- | ENST00000384125 | 8146120 | 30 |
| TRHR | ENST00000311762 | 8147877 | 30 |
| IL11RA | ENST00000378817 | 8154934 | 30 |
| MGC21881 /// LOC554249 | ENST00000377616 | 8155393 | 30 |
| ZNF483 | ENST00000358151 | 8157193 | 30 |
| C9orf169 | ENST00000400709 | 8159624 | 30 |
| MGC21881 /// LOC554249 | ENST00000377616 | 8161451 | 30 |
| --- | ENST00000364507 | 8168161 | 30 |
| CNR2 | ENST00000374472 | 7913705 | 25 |
| --- | ENST00000387003 | 7914137 | 25 |
| OIT3 | ENST00000334011 | 7928330 | 25 |
| --- | ENST00000388083 | 7929614 | 25 |
| --- | ENST00000365084 | 7934568 | 25 |
| FRG2 /// FRG2B /// FRG2C | ENST00000368515 | 7937251 | 25 |
| C14orf53 | ENST00000389594 | 7975154 | 25 |
| ODF3L1 | ENST00000332145 | 7985025 | 25 |
| FAM18A | ENST00000299866 | 7999412 | 25 |
| PRTN3 | ENST00000234347 | 8024048 | 25 |
| CFD | ENST00000327726 | 8024062 | 25 |
| TMED1 | ENST00000214869 | 8034101 | 25 |
| --- | ENST00000387150 | 8035937 | 25 |
| HSD17B14 | ENST00000263278 | 8038213 | 25 |
| BOK | ENST00000318407 | 8049876 | 25 |
| --- | ENST00000365609 | 8050801 | 25 |
| SNRPB | ENST00000381342 | 8064502 | 25 |
| EPHA6 | ENST00000338994 | 8081138 | 25 |
| SCARNA22 | NR_003004 | 8093576 | 25 |
| FLJ35424 | ENST00000404649 | 8093821 | 25 |
| BMP2K | ENST00000335016 | 8096004 | 25 |
| --- | ENST00000387555 | 8104723 | 25 |
| --- | ENST00000388664 | 8107115 | 25 |
| --- | ENST00000363365 | 8108566 | 25 |
| --- | ENST00000362861 | 8111358 | 25 |
| ZNF366 | ENST00000318442 | 8112584 | 25 |
| --- | ENST00000363181 | 8114581 | 25 |
| GRM6 | ENST00000319065 | 8116253 | 25 |
| LOC646093 | --- | 8116400 | 25 |
| HIST1H1E | ENST00000304218 | 8117377 | 25 |
| TIAM2 | ENST00000367174 | 8122933 | 25 |
| --- | ENST00000363074 | 8128712 | 25 |
| --- | ENST00000385777 | 8148331 | 25 |
| MTUS1 | ENST00000400046 | 8149500 | 25 |
| MUC21 | ENST00000383351 | 8177931 | 25 |
| WDR8 | ENST00000378322 | 7911839 | 20 |
| LOC100131195 | AK097743 | 7933190 | 20 |
| OR4D10 | ENST00000378245 | 7940182 | 20 |
| C12orf63 | ENST00000342887 | 7957688 | 20 |
| ELA1 | ENST00000293636 | 7963304 | 20 |
| DNAJC14 /// CIP29 | ENST00000317269 | 7963935 | 20 |
| FLJ40176 | ENST00000322527 | 7972670 | 20 |
| --- | ENST00000410207 | 7985308 | 20 |
| SYT17 | ENST00000396244 | 7993624 | 20 |
| PSME3 | ENST00000293362 | 8007397 | 20 |
| --- | ENST00000405656 | 8009515 | 20 |
| HN1 | ENST00000356033 | 8018305 | 20 |
| --- | ENST00000335523 | 8027385 | 20 |
| CYP2A7 /// CYP2A7P1 | ENST00000301146 | 8036981 | 20 |
| CALM2 | ENST00000272298 | 8052010 | 20 |
| ATXN10 | ENST00000252934 | 8073799 | 20 |
| ZMAT5 | ENST00000397779 | 8075276 | 20 |
| --- | ENST00000362493 | 8084215 | 20 |
| FHIT | ENST00000341848 | 8088458 | 20 |
| FRG2 /// FRG2B /// FRG2C | ENST00000368515 | 8104124 | 20 |
| SNX18 | ENST00000381410 | 8105328 | 20 |
| --- | ENST00000362433 | 8128445 | 20 |
| DTX2 | ENST00000307569 | 8133736 | 20 |
| ASB4 | ENST00000325885 | 8134376 | 20 |
| --- | ENST00000365242 | 8147445 | 20 |
| --- | ENST00000364204 | 8156450 | 20 |
| COL5A1 | ENST00000355306 | 8159142 | 20 |
| XK | ENST00000378616 | 8166723 | 20 |
| LCAP | ENST00000357566 | 8170786 | 20 |
| APOO | ENST00000379226 | 8171823 | 20 |
| PTPRU | ENST00000373779 | 7899562 | 15 |
| IL28RA | ENST00000327535 | 7913776 | 15 |
| NEUROG3 | ENST00000242462 | 7934083 | 15 |
| VAX1 | ENST00000277905 | 7936552 | 15 |
| ART4 | ENST00000228936 | 7961507 | 15 |
| LOC440131 | ENST00000400540 | 7968323 | 15 |
| C13orf31 | ENST00000325686 | 7968883 | 15 |
| ADAMTS7 | ENST00000388820 | 7990736 | 15 |
| --- | ENST00000332418 | 7997907 | 15 |
| SMTNL2 | ENST00000338859 | 8003892 | 15 |
| LOC284112 | AK098506 | 8012004 | 15 |
| ETV2 | ENST00000402764 | 8027920 | 15 |
| FUT2 | ENST00000391876 | 8030094 | 15 |
| C2orf39 | ENST00000288710 | 8040672 | 15 |
| LOC200383 /// DNAH6 | ENST00000237449 | 8043071 | 15 |
| ZFP36L2 | ENST00000282388 | 8051814 | 15 |
| --- | ENST00000385676 | 8055204 | 15 |
| CCDC108 | ENST00000341552 | 8059028 | 15 |
| --- | --- | 8065011 | 15 |
| C22orf27 | BC042980 | 8072400 | 15 |
| --- | ENST00000364444 | 8103041 | 15 |
| PDLIM3 | ENST00000284767 | 8104022 | 15 |
| --- | ENST00000330110 | 8104613 | 15 |
| --- | ENST00000384539 | 8107125 | 15 |
| GSTA3 | ENST00000370968 | 8127087 | 15 |
| COL21A1 | ENST00000370817 | 8127201 | 15 |
| --- | ENST00000390214 | 8130372 | 15 |
| MGC72080 | BC029615 | 8141169 | 15 |
| C9orf128 | ENST00000377984 | 8161154 | 15 |
| --- | ENST00000332418 | 8170322 | 15 |
| RGAG4 | NM_001024455 | 8173503 | 15 |
| PIP5K1A | ENST00000409426 | 7905365 | 10 |
| GPR161 | ENST00000367838 | 7922108 | 10 |
| --- | ENST00000385353 | 7925434 | 10 |
| OR56A3 | ENST00000329564 | 7938066 | 10 |
| OR5A2 | ENST00000302040 | 7948377 | 10 |
| WNT11 | ENST00000322563 | 7950534 | 10 |
| --- | --- | 7960259 | 10 |
| RAB37 | ENST00000340415 | 8009666 | 10 |
| LAIR1 | ENST00000391742 | 8039257 | 10 |
| --- | ENST00000388385 | 8041420 | 10 |
| CHAC2 | ENST00000295304 | 8041961 | 10 |
| --- | ENST00000387574 | 8062337 | 10 |
| --- | ENST00000387884 | 8062962 | 10 |
| BCL2L1 | ENST00000376062 | 8065569 | 10 |
| KDELR3 | ENST00000409006 | 8073015 | 10 |
| TMEM108 | ENST00000321871 | 8082767 | 10 |
| SPATA16 | ENST00000351008 | 8092187 | 10 |
| BTC | ENST00000395743 | 8101002 | 10 |
| SUPT3H | ENST00000371460 | 8126710 | 10 |
| EIF4B | ENST00000262056 | 8135268 | 10 |
| CHMP4C | ENST00000297265 | 8147057 | 10 |
| H2BFM | ENST00000243297 | 8169080 | 10 |
| --- | --- | 8180392 | 10 |
| NR5A2 | ENST00000367362 | 7908597 | 5 |
| FUCA1 | ENST00000374479 | 7913694 | 5 |
| --- | ENST00000386628 | 7925821 | 5 |
| TRIM49 | ENST00000332682 | 7939884 | 5 |
| MS4A6A | ENST00000323961 | 7948455 | 5 |
| C11orf10 | ENST00000257262 | 7948606 | 5 |
| HSPC152 | ENST00000308774 | 7949075 | 5 |
| RASAL1 | ENST00000261729 | 7966542 | 5 |
| --- | ENST00000387531 | 7975694 | 5 |
| PLDN | ENST00000220531 | 7983502 | 5 |
| PER1 | ENST00000354903 | 8012349 | 5 |
| AZU1 | ENST00000334630 | 8024038 | 5 |
| ALS2CR12 | ENST00000286190 | 8058203 | 5 |
| C20orf142 | ENST00000396825 | 8066407 | 5 |
| --- | ENST00000386848 | 8073680 | 5 |
| LOC100129113 | AK094477 | 8074307 | 5 |
| CERK | ENST00000216264 | 8076792 | 5 |
| --- | ENST00000385783 | 8083937 | 5 |
| PROS1 | ENST00000407433 | 8089015 | 5 |
| IL7R | ENST00000303115 | 8104901 | 5 |
| PCDHGA | ENST00000378105 | 8108757 | 5 |
| MUC3B /// MUC3A | ENST00000332750 | 8135015 | 5 |
| --- | ENST00000365355 | 8142534 | 5 |
| --- | --- | 8156969 | 5 |
| --- | ENST00000410626 | 8163013 | 5 |
| FAM47C | ENST00000358047 | 8166703 | 5 |
| NXF4 | ENST00000360035 | 8168940 | 5 |
| PIWIL4 | ENST00000299001 | 7943240 | 0 |
| --- | ENST00000384727 | 7968732 | 0 |
| ALDH6A1 | ENST00000350259 | 7980098 | 0 |
| TMEM64 | ENST00000324979 | 8151747 | 0 |
| --- | ENST00000364816 | 8168079 | 0 |

The table shows predictor genes in GRPS, identified by one-way ANOVA p-value filtering and Backward Elimination. When possible, the Ensembl transcript ID was used as gene identifier.

^1^Validation call frequency (%) describes the occurrence of each predictor transcript among the 20 biomarker signatures obtained by cross validation.
